# Supplementary material for: Oxaloacetate damages mitochondria by perturbing MIC60-dependent membrane remodeling
Source: PLoS Biol. 2026 Aug 28;24(8):e3003649. doi: 10.1371/journal.pbio.3003649 (PMC13537684; doi:10.1371/journal.pbio.3003649)

Source Data Fig 2.

H

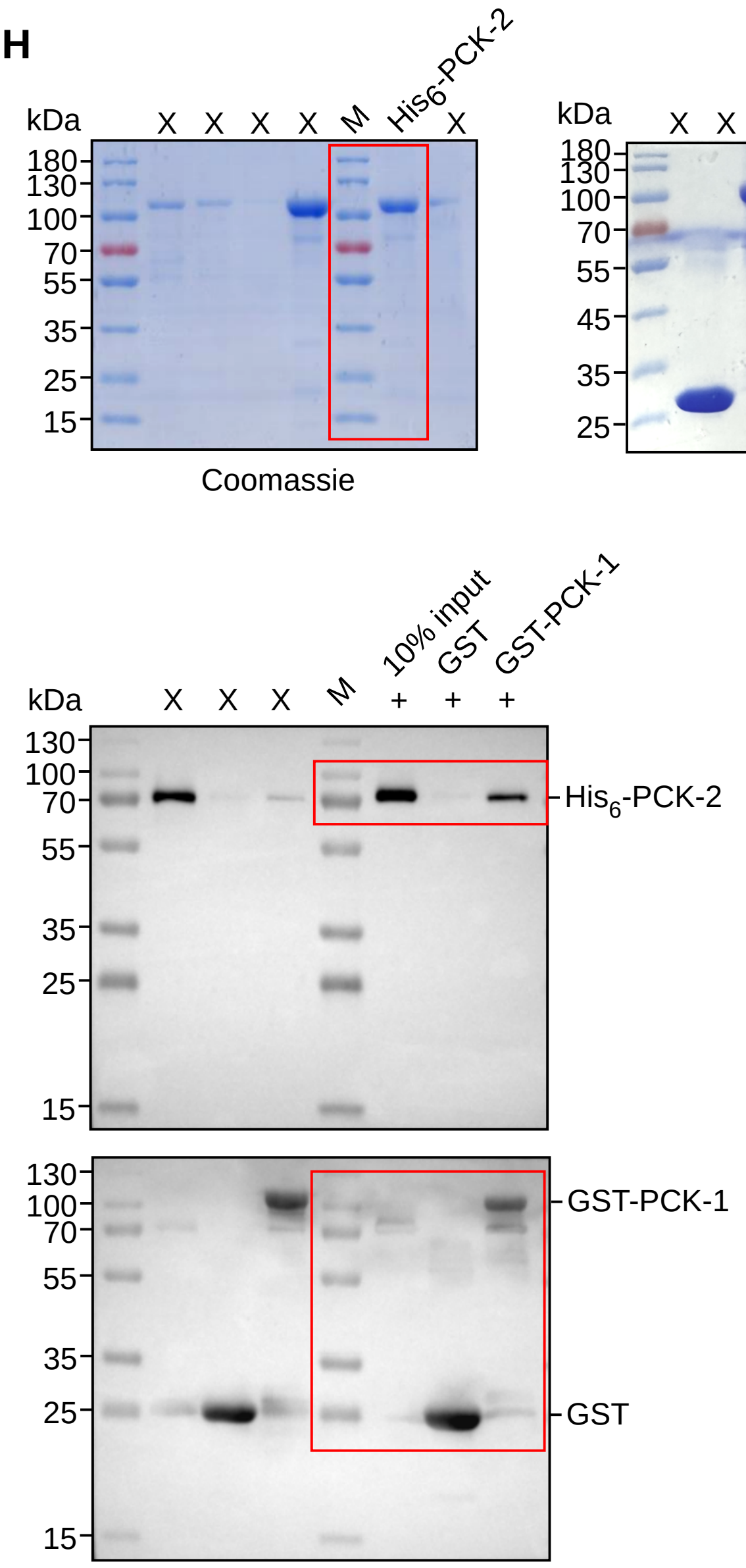

Source Data Fig 3.

D

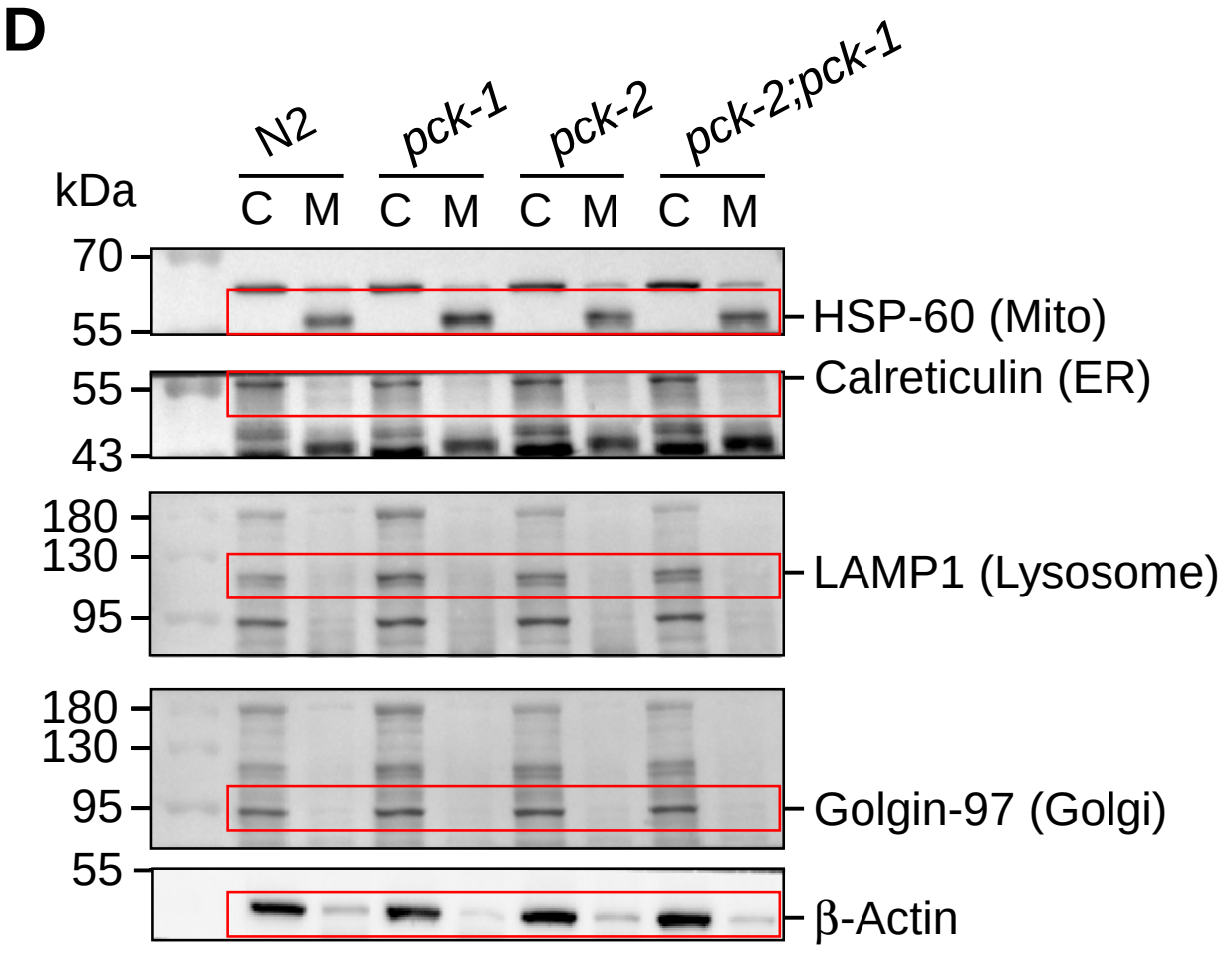

G

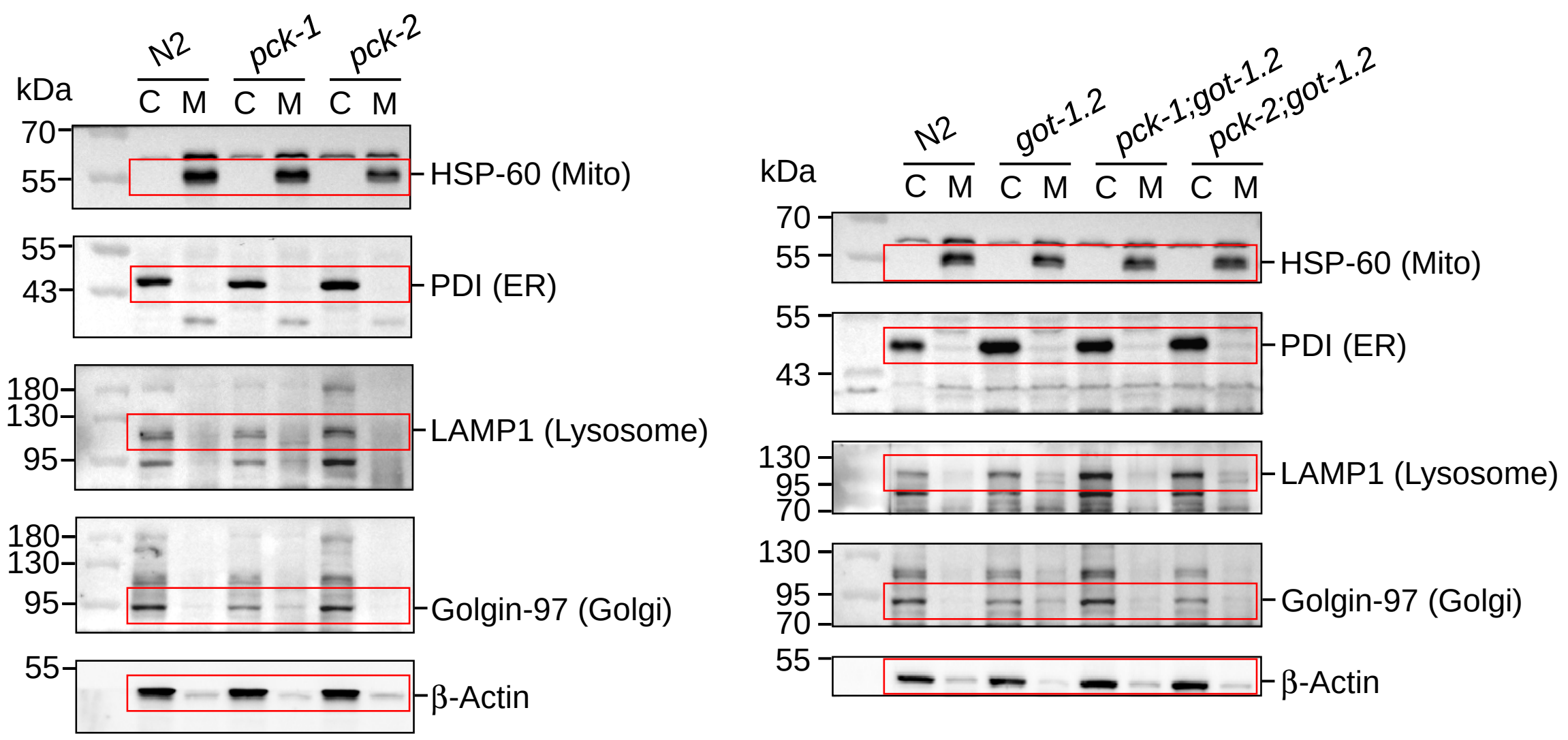

Source Data Fig 4.

A

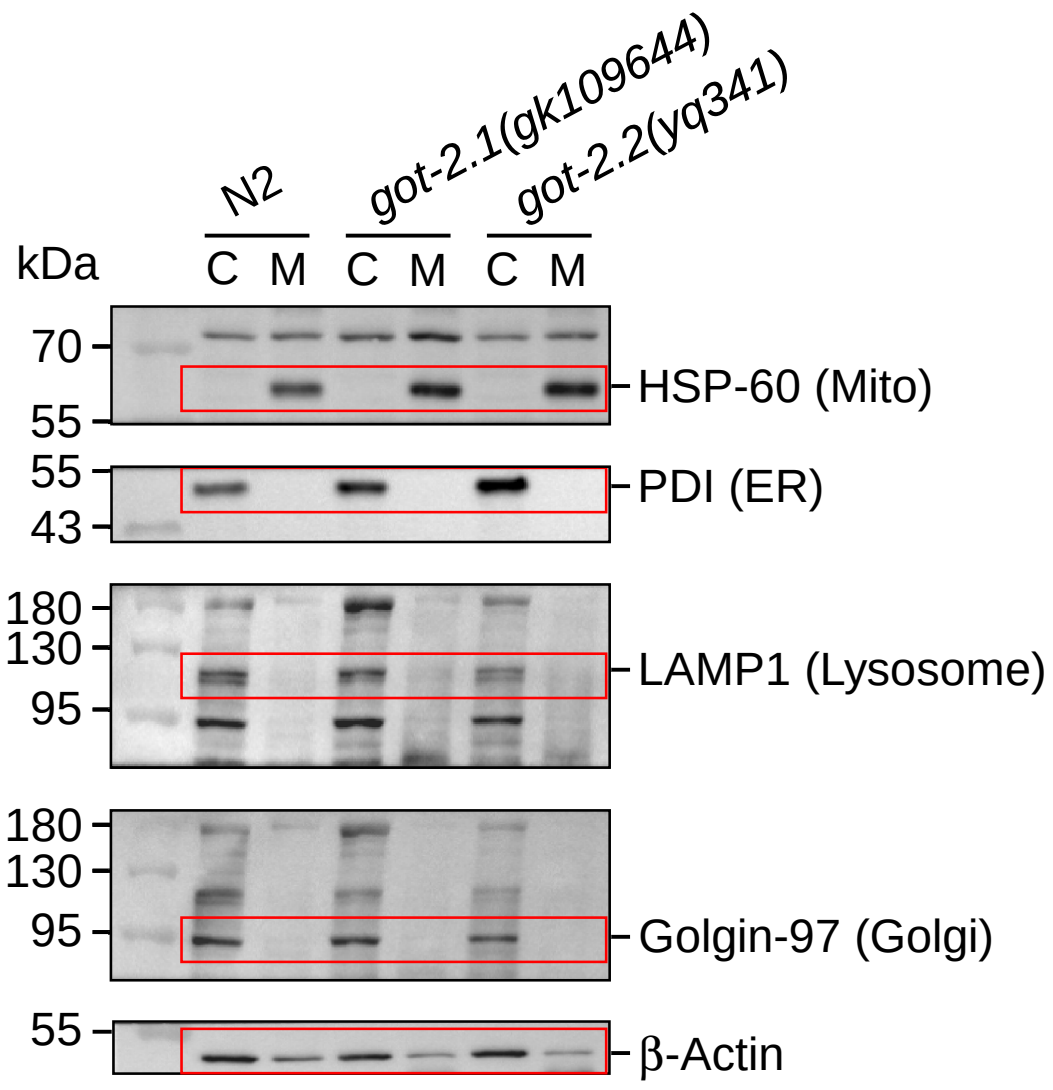

H

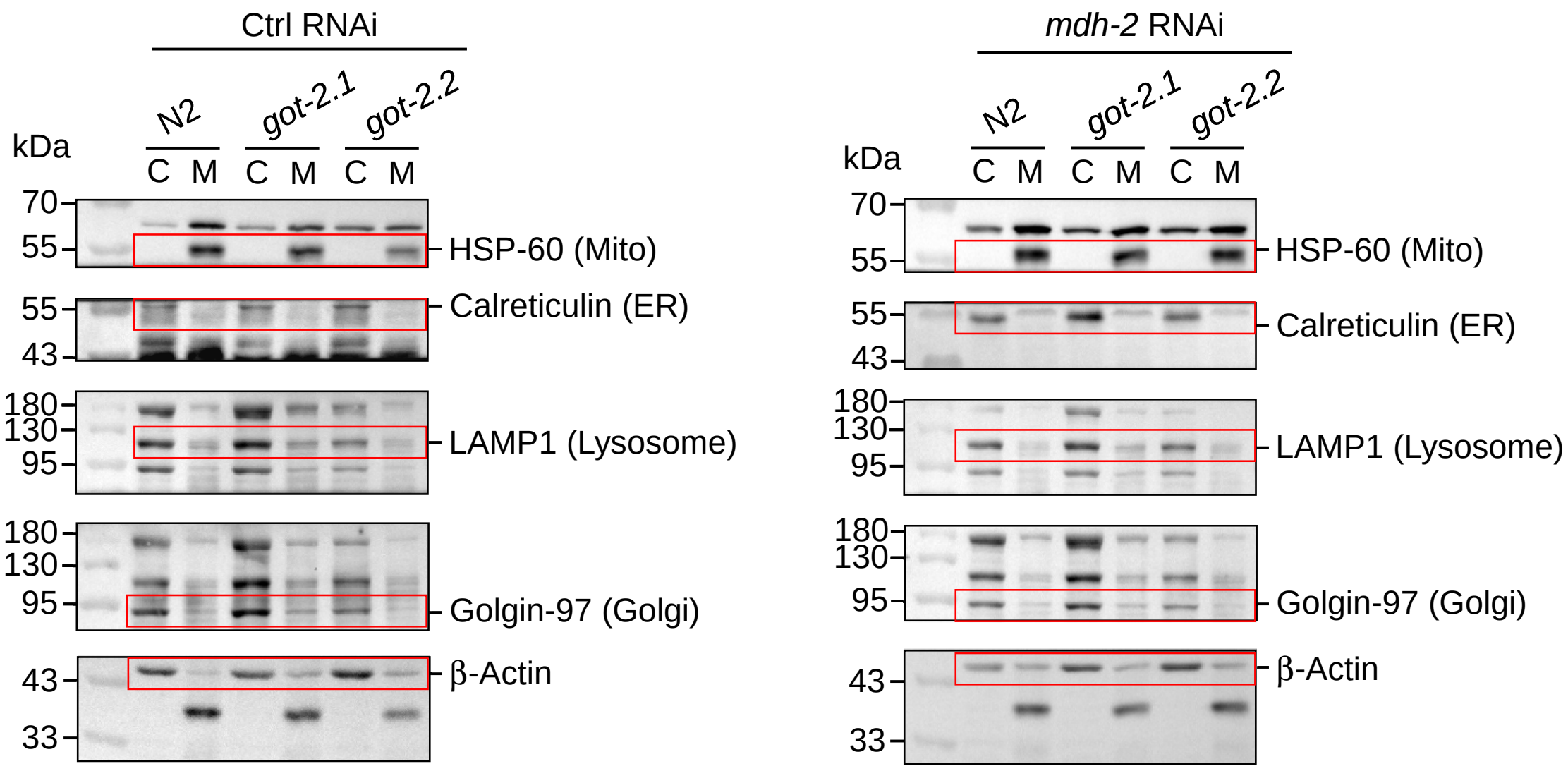

L

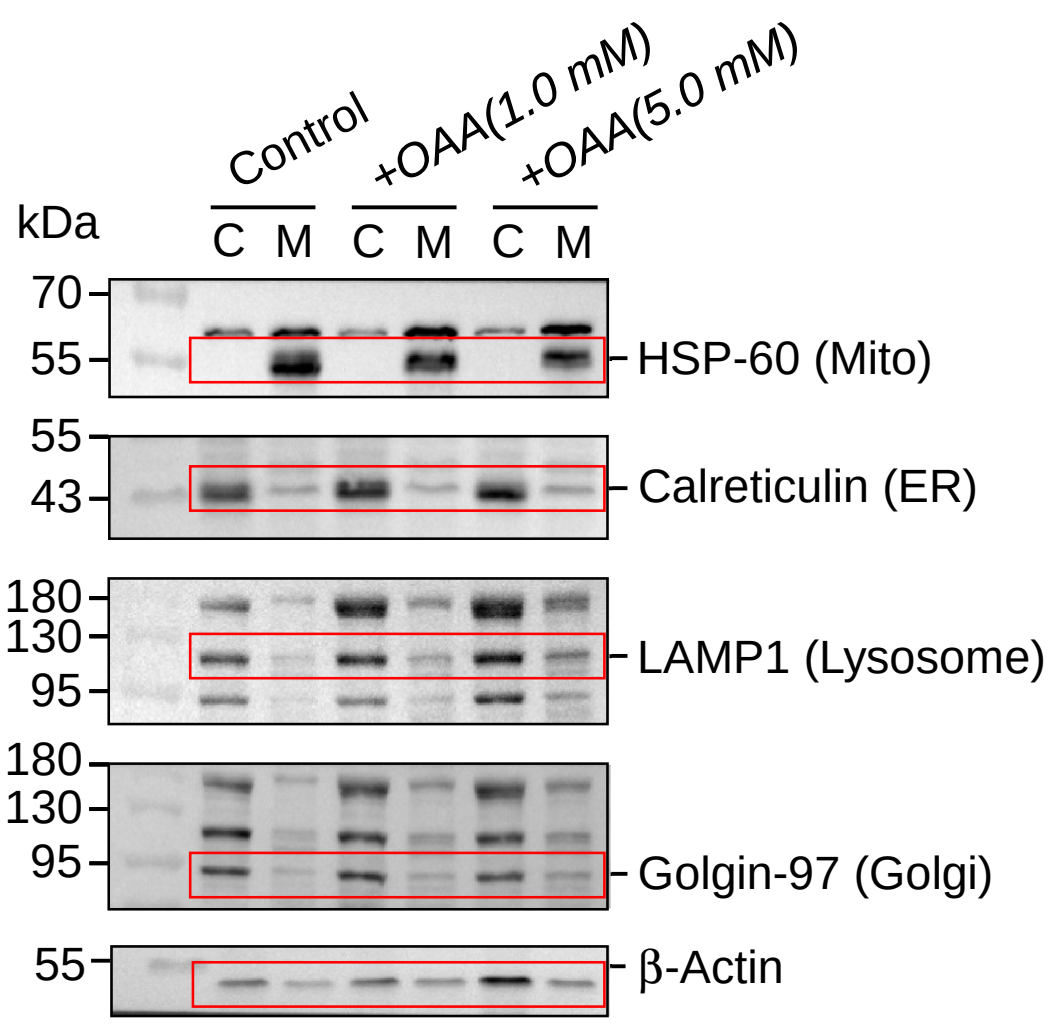

Source Data Fig 6.

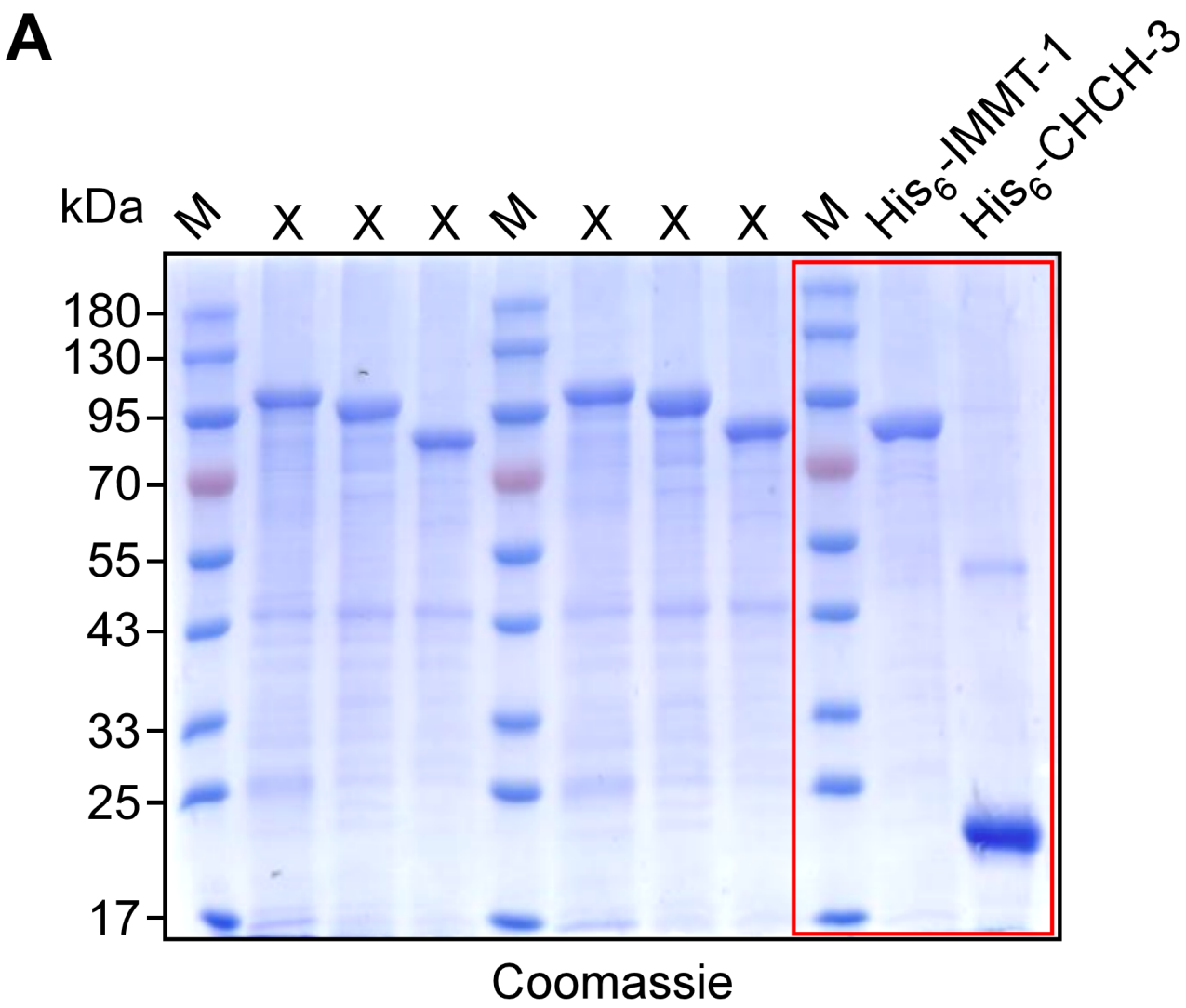

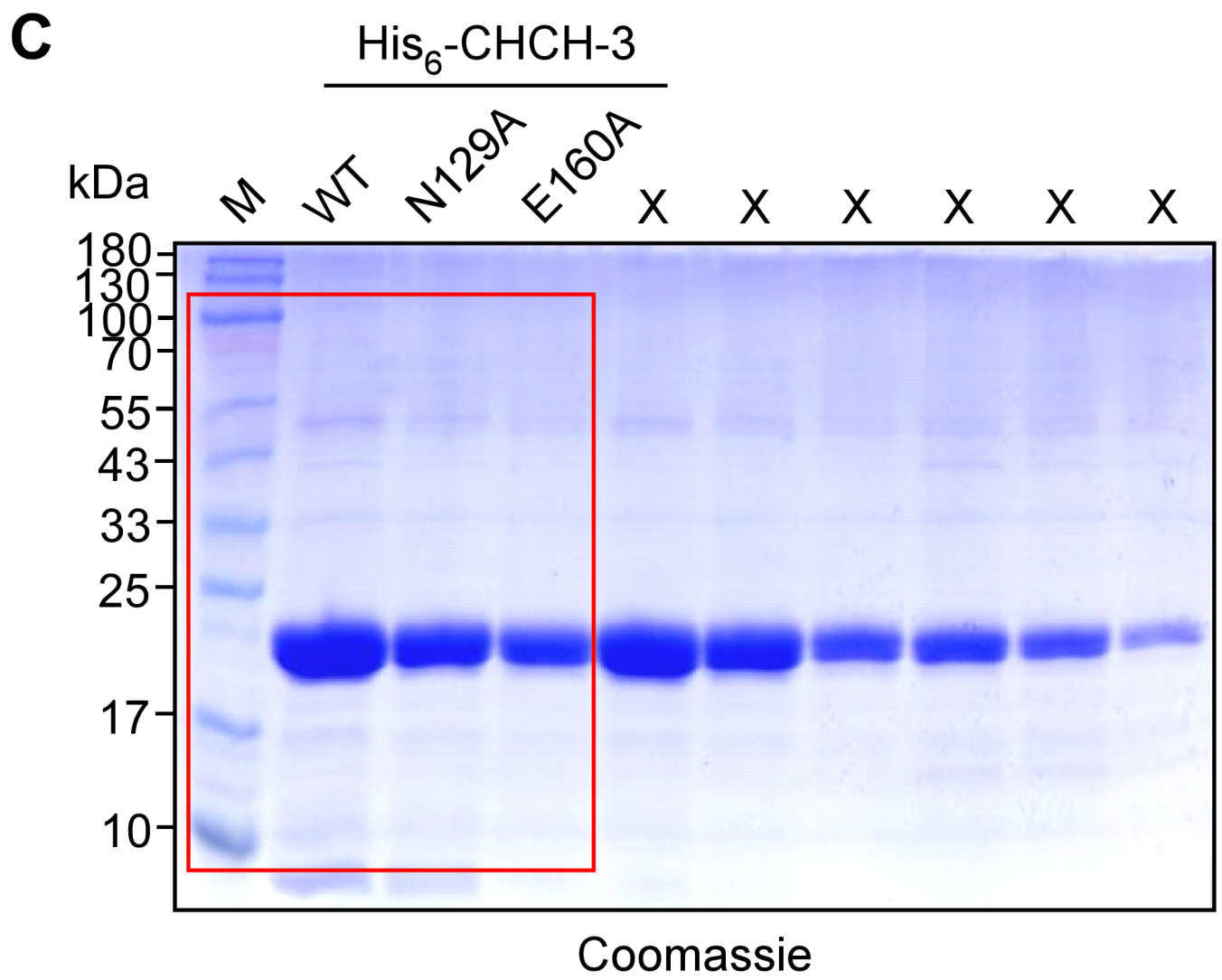

Source Data Figure 7

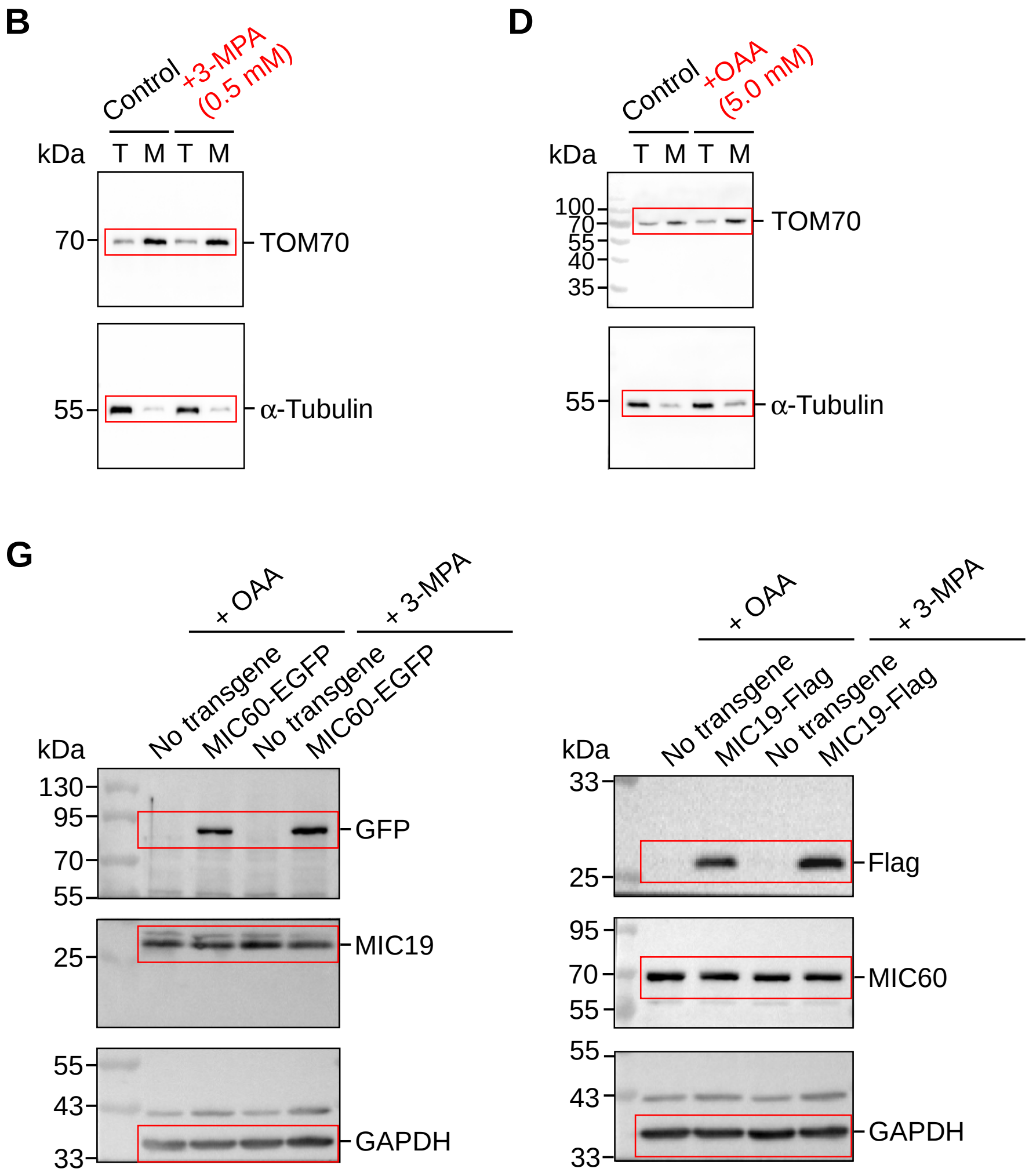

Source Data S6 Fig.

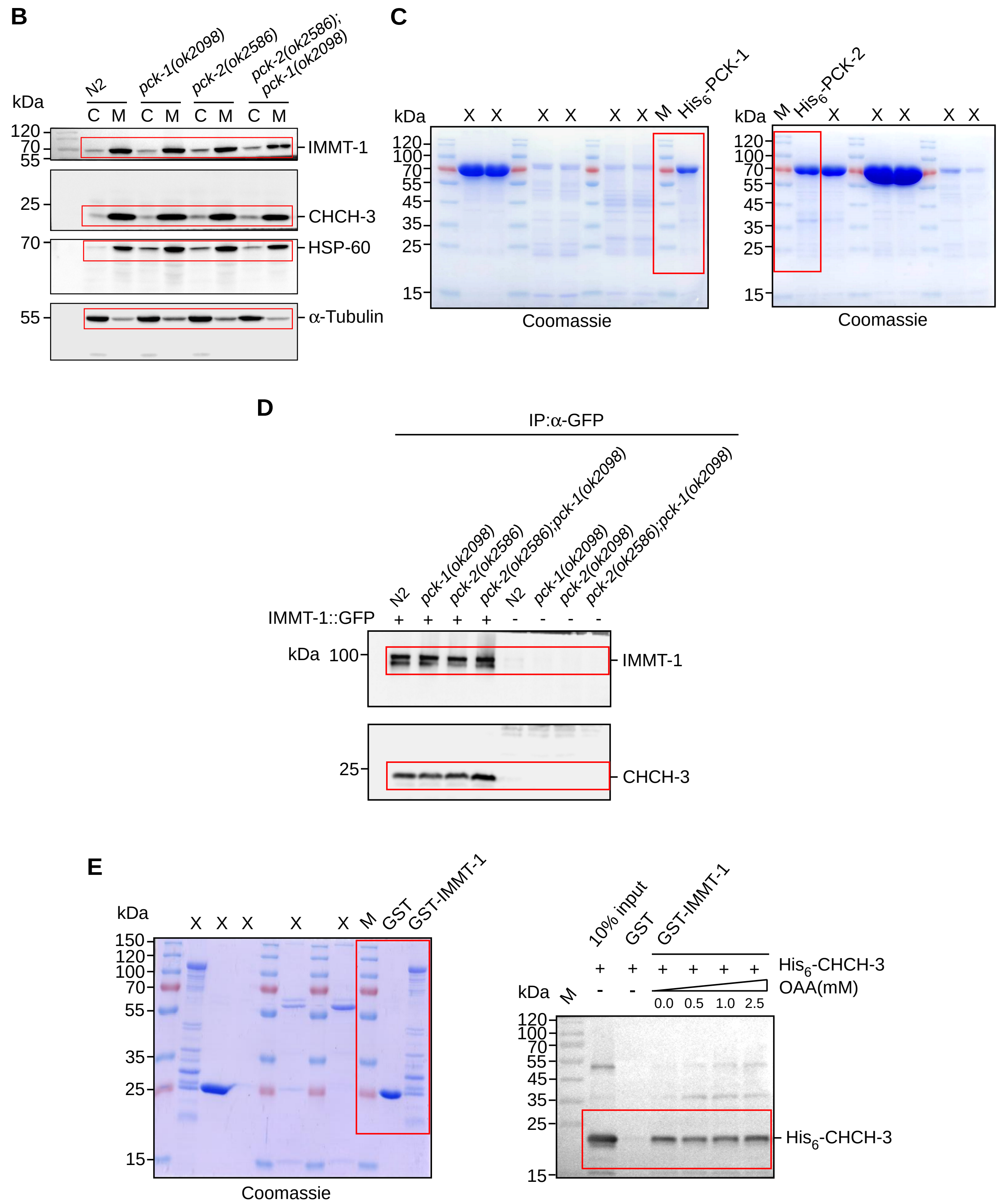

Supplement: Supplementary file 1 [file pbio.3003649.s012.pdf]
