# Supplementary material for: Oxaloacetate damages mitochondria by perturbing MIC60-dependent membrane remodeling
Source: PLoS Biol. 2026 Aug 28;24(8):e3003649. doi: 10.1371/journal.pbio.3003649 (PMC13537684; doi:10.1371/journal.pbio.3003649)
Supplement: Supplementary file 2 [file pbio.3003649.s008.docx]

**Supplementary Table 1. *Caenorhabditis elegans* strains used in this study.**

| **Strains** | **Type** | **Note** | **Sources** |
| --- | --- | --- | --- |
| *pck-2(yq270)* | Mutant | Loss of PCK-2 | This study |
| *pck-2(yq271)* | Mutant | Loss of PCK-2 | This study |
| *pck-2(ok2586)* | Mutant | Loss of PCK-2 | This study |
| *pck-1(ok2098)* | Mutant | Loss of PCK-1 | This study |
| *got-1.2(yq163)* | Mutant | Loss of GOT-1.2 | This lab |
| *got-2.1(gk109644)* | Mutant | Loss of GOT-2.1 | This study |
| *got-2.2(yq341)* | Knock out | Loss of GOT-2.2 | This study |
| *immt-1(yq160)* | Mutant | Loss of IMMT-1 | Reference 1 |
| *chch-3(tm2633)* | Mutant | Loss of CHCH-3 | Tokyo Women’s Medical University |
| *yq662(P_mdh-2_mdh-2::mKate)* | Knock in | Endogenous expression | This lab |
| *yq635(P_immt-1_immt-1::gfp)* | Knock in | Endogenous expression | This lab |
| *yq640(P_tomm-20_tomm-20::rfp::3×HA)* | Knock in | Endogenous expression | This lab |
| *yqIs157 (P_semo-1_mito::gfp)* | Integrated array | Overexpression | 1,2,3,4 |
| *yqIs179(P_semo-1_tomm-20::mCherry)* | Integrated array | Overexpression | 1,2,3,4 |
| *hqIs181(P_sdhl-1_mtLs::gfp)* | Integrated array | Overexpression | 1,2,3,4 |
| *yqEx2063(P_pck-2_pck-2::mCherry)* | Extrachromosomal array | Overexpression | This study |
| *yqEx2064(P_pck-1_pck-1::gfp)* | Extrachromosomal array | Overexpression | This study |
| *yqEx2065(P_pck-2_pck-2::gfp)* | Extrachromosomal array | Overexpression | This study |
| *yqEx2066(P_semo-1_hPCK1::gfp)* | Extrachromosomal array | Overexpression | This study |
| *yqEx2068(P_semo-1_hPCK2::gfp)* | Extrachromosomal array | Overexpression | This study |
| *yqEx2069(P_semo-1_hPCK2(△MTS)::gfp)* | Extrachromosomal array | Overexpression | This study |
| *yqEx2104(P_got-1.2_got-1.2::mCherry)* | Extrachromosomal array | Overexpression | This study |
| *yqEx2105(P_got-2.1_got-2.1::gfp)* | Extrachromosomal array | Overexpression | This study |
| *yqEx2106(P_got-2.2_got-2.2::gfp)* | Extrachromosomal array | Overexpression | This study |
| *yqEx1809(P_immt-1_immt-1::gfp)* | Extrachromosomal array | Overexpression | This study |
| *yqEx2189(P_chch-3_chch-3)* | Extrachromosomal array | Overexpression | This study |
| *yqEx2190(P_chch-3_chch-3(N129A))* | Extrachromosomal array | Overexpression | This study |
| *yqEx2191(P_chch-3_chch-3(E160A))* | Extrachromosomal array | Overexpression | This study |
| *yqIs307(P_fzo-1_fzo-1::gfp)* | Integrated array | Overexpression | This study |
| *yqEx1802(P_eat-3_eat-3::gfp)* | Extrachromosomal array | Overexpression | This study |
| *yqEx1708(P_F54A3.5_F54A3.5(CDS)::mKate)* | Extrachromosomal array | Overexpression | This study |
| *yqEx1713(P_col-19_moma-1::mKate)* | Extrachromosomal array | Overexpression | This study |
| *yqEx1710(P_col-19_W04C9.2::mKate)* | Extrachromosomal array | Overexpression | This study |
| *yqEx1805(P_nuo-3_nuo-3::gfp)* | Extrachromosomal array | Overexpression | This study |
| *yqEx1806(P_nuo-4_nuo-4::gfp)* | Extrachromosomal array | Overexpression | This study |
| *yqEx1816(P_sdha-2_sdha-2::gfp)* | Extrachromosomal array | Overexpression | This study |
| *yqEx1812(P_atp-2_atp-2::gfp)* | Extrachromosomal array | Overexpression | This study |
