# Supplementary material for: Oxaloacetate damages mitochondria by perturbing MIC60-dependent membrane remodeling
Source: PLoS Biol. 2026 Aug 28;24(8):e3003649. doi: 10.1371/journal.pbio.3003649 (PMC13537684; doi:10.1371/journal.pbio.3003649)
Supplement: Supplementary file 3 [file pbio.3003649.s009.docx]

**Supplementary Table 2. Expression vectors.**

| **Vectors** | **Promoter (insertion sites)** | **Gene (insertion sites)** | **Backbone** |
| --- | --- | --- | --- |
| P*_semo-1_mito::gfp* | *semo-1* promoter | *mito-gfp* | pPD49.26 |
|  | (3Kb;BamHI) | (NheI-KpnI) |  |
| P*_semo-1_tomm-20::mCherry* | *semo-1* promoter | *tomm-20-mCh* | pPD49.26 |
|  | (3Kb;BamHI) | (NheI-KpnI) |  |
| P*_pck-2_pck-2::mCherry* | *pck-2* promoter | *pck-2* gDNA | pPD49.26-mCh |
|  | (2Kb;BamHI) | (SalI-NheI) |  |
| P*_pck-1_pck-1::gfp* | *pck-1* promoter | *pck-1* gDNA | pPD49.26-GFP |
|  | (2Kb;SalI) | (BamHI) |  |
| P*_pck-2_pck-2::gfp* | *pck-2* promoter | *pck-2* gDNA | pPD49.26-GFP |
|  | (2Kb;SalI) | (BamHI) |  |
| P*_semo-1_hPCK1::gfp* | *semo-1* promoter | *hPCK1* CDS | pPD49.26-GFP |
|  | (3Kb;PstI-BamHI) | (BamHI-NheI) |  |
| P*_semo-1_hPCK2::gfp* | *semo-1* promoter | *hPCK2* CDS | pPD49.26-GFP |
|  | (3Kb;PstI-BamHI) | (BamHI-NheI) |  |
| P*_semo-1_hPCK2(△MTS)::gfp* | *semo-1* promoter | *hPCK2(△MTS)* CDS | pPD49.26-GFP |
|  | (3Kb;PstI-BamHI) | (BamHI-NheI) |  |
| P*_got-1.2_got-1.2::mCherry* | *got-1.2* promoter | *got-1.2* gDNA | pPD49.26-mCh |
|  | (2Kb;BamHI) | (SalI-NheI) |  |
| P*_got-2.1_got-2.1::gfp* | *got-2.1* promoter | *got-2.1* gDNA | pPD49.26-GFP |
|  | (2Kb;SalI) | (BamHI) |  |
| P*_got-2.2_got-2.2::gfp* | *got-2.2* promoter | *got-2.2* gDNA | pPD49.26-GFP |
|  | (2Kb;SalI) | (BamHI) |  |
| P*_immt-1_immt-1::gfp* | *immt-1* promoter | *immt-1* gDNA | pPD49.26-GFP |
|  | (2Kb;SphI) | (XbaI) |  |
| P*_chch-3_chch-3* | *chch-3* promoter | *chch-3* gDNA | pPD49.26 |
|  | (2Kb;SphI) | (XbaI) |  |
| P*_chch-3_chch-3(N129A)* | *chch-3* promoter | *chch-3* cDNA | pPD49.26 |
|  | (2Kb;SphI) | (SacI) |  |
| P*_chch-3_chch-3(E160A)* | *chch-3* promoter | *chch-3* cDNA | pPD49.26 |
|  | (2Kb;SphI) | (SacI) |  |
| P*_fzo-1_fzo-1::gfp* | *fzo-1* promoter | *fzo-1* gDNA | pPD49.26-mKate |
|  | (2Kb;HindIII) | (SalI-BamHI) |  |
| P*_eat-3_eat-3::gfp* | *eat-3* promoter | *eat-3* gDNA | pPD49.26-mKate |
|  | (2Kb;SalI) | (BamHI) |  |
| P*_F54A3.5_F54A3.5::mKate* | *F54A3.5* promoter | *F54A3.5* CDS | pPD49.26-mKate |
|  | (2Kb;SalI) | (BamHI) |  |
| P*_col-19_moma-1::mKate* | *col-19* promoter | *moma-1* gDNA | pPD49.26-GFP |
|  | (2Kb;SalI-BamHI) | (BamHI-NheI) |  |
| P*_col-19_W04C9.2::mKate* | *col-19* promoter | *W04C9.2* gDNA | pPD49.26-GFP |
|  | (2Kb;SalI-BamHI) | (BamHI-NheI) |  |
| P*_nuo-3_nuo-3::gfp* | *nuo-3* promoter | *nuo-3* gDNA | pPD49.26-GFP |
|  | (2Kb;SalI) | (BamHI) |  |
| P*_nuo-4_nuo-4::gfp* | *nuo-4* promoter | *nuo-4* gDNA | pPD49.26-GFP |
|  | (2Kb;SalI) | (BamHI) |  |
| P*_sdha-2_sdha-2::gfp* | *sdha-2* promoter | *sdha-2* gDNA | pPD49.26-GFP |
|  | (2Kb;SalI) | (BamHI) |  |
| P*_atp-2_atp-2::gfp* | *atp-2* promoter | *atp-2* gDNA | pPD49.26-GFP |
|  | (2Kb;SalI) | (BamHI) |  |
| pET21a-His_6_-PCK-2 | T7 promoter | *pck-2* CDS | pET21a |
|  |  | (EcoRI-HindIII) |  |
| pET28a-His_6_-IMMT-1 | T7 promoter | *immt-1* CDS | pET28a |
|  |  | (EcoRI-HindIII) |  |
| pET21a-His_6_-CHCH-3 | T7 promoter | *chch-3* CDS | pET21a |
|  |  | (EcoRI-HindIII) |  |
| pET21a-His_6_-CHCH-3(N129A) | T7 promoter | *chch-3(N129A)* CDS | pET21a |
|  |  | (EcoRI-HindIII) |  |
| pET21a-His_6_-CHCH-3(E160A) | T7 promoter | *chch-3(E160A)* CDS | pET21a |
|  |  | (EcoRI-HindIII) |  |
| pGEX4T-1-PCK-1 | Tac promoter | *pck-1* CDS | pGEX4T-1 |
|  |  | (EcoRI-SalI) |  |
| pGEX4T-1-IMMT-1 | Tac promoter | *immt-1* CDS | pGEX4T-1 |
|  |  | (EcoRI-SalI) |  |
| pEGFP-N1-hPCK1 | CMV promoter | *hPCK1* CDS | pEGFP-N1 |
|  |  | (EcoRI-XmaI) |  |
| pEGFP-N1-hPCK2 | CMV promoter | *hPCK2* CDS | pEGFP-N1 |
|  |  | (EcoRI-XmaI) |  |
| pEGFP-N1-MIC60 | CMV promoter | *MIC60* CDS | pEGFP-N1 |
|  |  | (EcoRI-XmaI) |  |
| pCMVTag2B-MIC19 | CMV promoter | *MIC19* CDS | pCMV-Tag2B |
|  |  | (SacI-NotI) |  |
