# Supplementary material for: Oxaloacetate damages mitochondria by perturbing MIC60-dependent membrane remodeling
Source: PLoS Biol. 2026 Aug 28;24(8):e3003649. doi: 10.1371/journal.pbio.3003649 (PMC13537684; doi:10.1371/journal.pbio.3003649)
Supplement: Supplementary file 4 [file pbio.3003649.s010.docx]

**Supplementary Table 3. Antibodies and reagents used in this study.**

| **Antibodies** | **Antigen Species** | **Sources** | **Identifier** |
| --- | --- | --- | --- |
| Mouse monoclonal anti-6×His-tag | Species independent | proteintech | 66005-1-Ig |
| Mouse monoclonal anti-GST-tag | Species independent | proteintech | 66001-2-Ig |
| Mouse monoclonal anti-HSP-60 | *C. elegans* | DHSB | AB_10572252 |
| Mouse monoclonal anti-α-Tubulin | Mouse, Human, Rat, Chicken, Bovine, Yeast and *C. elegans* | Sigma-Aldrich | T6199 |
| Mouse monoclonal anti-β-Actin | Mouse, Rat, Human, Drosophila, Yeast and *C. elegans* | Santa Cruz | sc-517582 |
| Mouse monoclonal anti-IMMT-1 | *C. elegans*, IMMT-1 (100-500 aa) | Homemade | Reference 1 |
| Mouse monoclonal anti-CHCH-3 | *C. elegans*, Full length CHCH-3 | Homemade | Reference 2 |
| Mouse monoclonal anti-TOM70 | Human, Mouse, Rat, Pig | proteintech | 66593-1-Ig |
| Goat Anti-Mouse IgG H&L (HRP) | Mouse | Abcam | ab205719 |
| Goat Anti-Rabbit IgG H&L (HRP) | Rabbit | Abcam | ab6721 |
| LAMP1 | Human | Cell Signaling Technology | D4O1S |
| Golgin-97 | Human, Mouse | Cell Signaling Technology | D8P2K |
| Calreticulin | Human, Mouse, Rat | Cell Signaling Technology | D3E6 |
| PDI | Human, Mouse, Rat, Pig | proteintech | 66422-1-Ig |
| Flag | Species independent | Sigma-Aldrich | F7425 |
| GFP | Species independent | Sigma-Aldrich | AB3080 |
| MIC19 | Human, Mouse, Rat | proteintech | 25625-1-AP |
| MIC60/Mitofilin | Human | Abcam | ab264372 |
| GAPDH | Human, Mouse, Rat, Pig, Zebrafish, Yeast | proteintech | 60004-1-Ig |
| Goat anti-Mouse IgG-Adsorbed Secondary Antibody, Alaxa Fluor 488 | Species independent | Life Technologies | A11001 |
| Goat anti-Rabbit IgG-Adsorbed Secondary Antibody, Alaxa Fluor 488 | Species independent | Life Technologies | A11034 |
| Goat anti-Mouse IgG-Adsorbed Secondary Antibody, Alaxa Fluor 568 | Species independent | Life Technologies | A11031 |
| **Reagents** | | **Sources** | **Identifier** |
| ATP Assay Kit | | Beyotime | S0026 |
| Carbonyl cyanide 4-(trifluoromethoxy)phenylhydrazone) (FCCP) | | MedChem Express | HY-100410 |
| Agilent Seahorse XF Calibrant (pH 7.4) | | Agilent Technologies | 100840-000 |
| IPTG (Isopropyl β-D-thiogalactoside) | | MedChem Express | [367-93-1](https://www.medchemexpress.cn/cas/367-93-1.html) |
| Imidazole | | Sigma-Aldrich | 56750 |
| cOmplete^TM^, EDTA-free Protease Inhibitor Cocktail | | Roche | 4693132001 |
| Chelating Sepharose^TM^ Fast Flow | | GE Healthcare | 17-0575-01 |
| Glutathione Sepharose^TM^ 4B | | GE Healthcare | 17-0756-01 |
| GFP-Nanoab-Agarose Beads | | Lablead | GNA-50-1000 |
| Oxaloacetate Assay Kit | | Sigma-Aldrich | MAK070 |
| Oxaloacetate (OAA) | | Sigma-Aldrich | O4126 |
| Aspartate (Asp) | | Sigma-Aldrich | [A6558](https://www.sigmaaldrich.cn/CN/zh/product/sigma/a6558) |
| Malate (Mal) | | MedChem Express | [6915-15-7](https://www.medchemexpress.cn/cas/6915-15-7.html) |
| 3-Mercaptopicolinic Acid (3-MPA) | | ApexBio | C3543 |
| TRIzol Reagent | | ThermoFisher | 15596018CN |
| Reverse Transcription System | | Promega | A3800 |
| SYBR Green qPCR Master Mix | | MedChem Express | HY-K0501 |
| His-Tag Labeling Kit RED-tris-NTA 2nd Generation | | NanoTemper | MO-L018 |
| Folch Fraction I | | Sigma-Aldrich | B1502 |
| Lipofectamine 2000 | | Life Technologies | 11668019 |
| MitoTracker™ Red CMXRos | | ThermoFisher | M7512 |
| Pierce™ Anti-HA Magnetic Beads | | ThermoFisher | 88837 |
| DTT | | Sigma-Aldrich | 10708984001 |
| H_2_O_2_ | | Sigma-Aldrich | 88597 |
| Triton X-100 | | Solarbio | T8200 |
| Methyl cellulose | | MedChem Express | [9004-67-5](https://www.medchemexpress.cn/cas/9004-67-5.html) |

References

1. Zhou, J., et al. (2022). A feedback loop engaging propionate catabolism intermediates controls mitochondrial morphology. Nature Cell Biology. 24: 526-537.
2. Bai, Y., et al*.* (2025). Mitochondria-associated condensates maintain mitochondrial homeostasis and promote lifespan. Nature Aging. 5: 1983–2002.
