# Supplementary material for: Oxaloacetate damages mitochondria by perturbing MIC60-dependent membrane remodeling
Source: PLoS Biol. 2026 Aug 28;24(8):e3003649. doi: 10.1371/journal.pbio.3003649 (PMC13537684; doi:10.1371/journal.pbio.3003649)
Supplement: Supplementary file 5 [file pbio.3003649.s011.docx]

**Supplementary Table 4. Oligonucleotides used for qRT-PCR in this study.**

| **Oligonucleotides** | **Forward primer (5'-3')** | **Reverse primer (5'-3')** | **Product (bp)** |
| --- | --- | --- | --- |
| *chch-3* | AGCATCCCAGAGTGCAGAACAA | AACTCTGGTCGCATTGACTCG | 130 |
| *immt-1* | GTGGATGTTCGCAAAGATGTGAC | TCGCATTGATGGTCTTCAGCTTG | 163 |
| *F54A3.5* | TCTCGTAGTGAAGATGAAGTCGGA | GACGCCTGATCCAAACCAAATTG | 150 |
| *W04C9.2* | CGGTGGAAAGTGGAACAGTGCT | GTCGATTGGCCACTGTGTTCAC | 86 |
| *moma-1* | ATTCTGGCAACTTGTCACCTCG | TCCTGCTCACATGCGATACGAA | 180 |
