# Supplementary material for: Oxaloacetate damages mitochondria by perturbing MIC60-dependent membrane remodeling
Source: PLoS Biol. 2026 Aug 28;24(8):e3003649. doi: 10.1371/journal.pbio.3003649 (PMC13537684; doi:10.1371/journal.pbio.3003649)
Supplement: Supplementary file 6 [file pbio.3003649.s016.docx]

**Point-by-point responses to the reviewers’ comments**

**Reviewer #1:**

In this manuscript, the authors investigate the impact of oxaloacetate (OAA) accumulation on mitochondrial disfunction in C. elegans, unveiling a physical interaction between OAA and IMMT-1/MIC60 proteins. Using an unbiased genetic screening in C. elegans, they identified two mutants in the pck-2 gene with abnormally spherical and enlarged mitochondria compared to N2 wild-type animals. pck-2 encodes for an enzyme that, together with PCK-1, converts cytosolic OAA into PEP and both pck-1, pck-2 and pck1;pck2 double mutants display disrupted mitochondria, reduced ATP levels, reduced OCR and reduced body length, body bends and lifespan compared to wild-type animals. This phenotype was associated with increased cytosolic and mitochondrial OAA levels and mutation of got-1.2 or silencing of mdh-2 both rescued mitochondrial morphology and normal OAA levels, as these two enzymes respectively produce OAA in the cytosol and in the mitochondria. On the other hand, got-2.1 and got-2.2 mutants are no longer able to convert mitochondrial OAA into Aspartate, leading to augmented mitochondrial OAA levels and to mitochondrial disfunction. Authors then tested the overexpression of different proteins involved in membrane remodeling in pck-1 and pck-2 mutants to find possible players involved in the observed mitochondrial abnormalities. They found out that overexpression of both IMMT-1/MIC60 and CHCH-3/MIC19 restored normal mitochondrial morphology and counteracted the effects of increased OAA levels, with IMMT-1 acting downstream of CHCH-3. Mechanistically, they demonstrated that only CHCH-3 is able to bind OAA and that this binding CHCH-3 function of promoting IMMT-1- dependent membrane shaping and mitochondrial tubulation. Finally, authors showed that these mechanisms are conserved also in human cells, as the pharmacological inhibition of hPCK1 and hPCK2 leads to OAA increase and so to abnormal mitochondrial morphology and this phenotype is rescued by overexpression of both MIC60 and MIC19. The work presented in this paper is linear and well organized. The rationale behind each experiment is well explained and conclusions drawn generally make sense. In all, this manuscript advances the knowledge of how the elevation of a single metabolite can impact mitochondrial morphology (and so cell functions) acting through a newly discovered MIC19/MIC60-dependend axis. The experiments are presented in a logical flow, however some concerns stand.

**Response**: Thank you very much for appreciating our findings! We are grateful to your excellent and constructive suggestions and comments. Following these suggestions, we have performed additional experiments and supplied new data in our revision, which greatly improved the quality of our study. Listed below are our point-by-point responses to your suggestions.

1. My main, fundamental, concern is that authors do not robustly demonstrate whether OAA accumulation occurs in the cytoplasm or in the mitochondria. They quantify both cytosolic and mitochondrial OAA post fractionation, but metabolites diffuse rapidly and their compartmentalization cannot be assessed by standard organelles partitioning. This is very well known. Authors should add more controls to their experiments (e.g.: quantify metabolites that are exclusively mito or cyto, in parallel; or introduce some type of internal standard for organelle separation e.g.:PMID: 34856123). Or use orthogonal approaches that enable spatial resolution.

**Response**: These are excellent suggestions! Our original data suggest that OAA accumulates in both cytosol and mitochondria in single and double mutants of *pck-1* and *pck-2*. The cytosolic and mitochondrial fractions were prepared by classic differential centrifugation and the results were robustly reproducible. Following your suggestions, we re-performed the analysis of OAA levels by isolating mitochondrial fractions with affinity purification. To do this, we crossed an *yq640* allele into single or double mutants of *pck-1*, *pck-2*, double mutants of *got-1.2* with *pck-1* or *pck-2*, and single mutants of *got-2.1* and *got-2.2*. *yq640* was generated by CRISPR/Cas9-mediated knock-in and expressed TOMM-20::RFP::3xHA at endogenous level, allowing us to isolate mitochondria with 3xHA-based affinity purification. With this, we prepared cytosolic and mitochondrial fractions, and used antibodies of multiple organelle-specific proteins, including HSP-60 (mitochondrion), Calreticulin or PDI (ER), LAMP1 (lysosome), Golgin-97 (Golgi), and β-Actin, to examine the purity of mitochondrial fraction. By western blotting analysis, we determined that the mitochondrial fractions were well separated from cytosolic ones. Our new results indicated that both cytosolic and mitochondrial OAA levels were increased in single and double mutants of *pck-1* and *pck-2* (please see below in **Fig 3. D**), but were suppressed by loss of *got-1.2* (please see below in **Fig 3. G**). The mitochondrial OAA levels were increased in *got-2.1* and *got-2.2* mutants (please see below in **Fig 4. A**), but were suppressed by *mdh-2* RNAi (please see below in **Fig 4. H**). In addition, supplementation of exogenous OAA indeed caused increase in both cytosolic and mitochondrial OAA levels (please see below in **Fig 4. L**). These new results are consistent with the previous data in our original submission. We have now presented these new results, with additional organelle-specific markers in western blots, in our revised manuscript. We have also added description of mitochondrial purification method and the information about these TOMM-20::RFP::3xHA-expressing strains in our revised paper. We hope you will agree with us on these revisions.

2. Authors tend to be very loose in the interpretation of data the point to a specific elevation of the mito OAA pool. First, the original hit (mutant pck-2) increases cytoplasmic OAA. Second, TCA and GOT enzymes are bi-directional. Third, in the final experimentation (on human cells) rescue is performed through extracellular OAA supplementation, that would feed the cytoplasmic pool and only very indirectly the mito pool. Only to mention a few. I think authors should be more direct in their analysis. At the end of the day, I understand the challenge posed by the matter. But lacking a very robust quantification of metabolite compartmentalization, conclusions must be very judicious.

**Response**: We understand these points and totally agree with you that we need to be very judicious in drawing conclusions. As you pointed out, it remains to be extremely challenging to accurately determine the compartmentalization of metabolites. In our study, we made all efforts to avoid any non-objective assumption by combining genetic analysis, mitochondrial defects and metabolite analysis to reveal the adverse effect of OAA accumulation on mitochondria. As you can see from our results from **Fig. 4** (please see below and the revised manuscript), loss of *got-2.1* or *got-2.2* caused mitochondrial OAA accumulation and mitochondrial fragmentation (**Fig 4. A** and **I**), which is similar to loss of *pck-1* and *pck-2*. RNAi of *mdh-2* suppressed the accumulation of mitochondrial OAA, and consequently the spherical and fragmented mitochondria were restored to tubular and connected morphology (**Fig 4. H** and **I**). In addition, exogenously supplemented OAA, but not Asp or Mal, caused similar mitochondrial defects, evidenced by fluorescence imaging and TEM analysis (**Fig 4. J** and **K**). The exogenously supplemented OAA caused a significant increase in both cytosolic and mitochondrial OAA levels (**Fig 4. L**). The positive correlation of mitochondrial OAA levels and mitochondrial defects, as well as other experimental data in this research, led us to conclude that mitochondrial accumulation of OAA damages mitochondria. We hope that you will agree with us on these points, and it is indeed our on-going and future effort to develop more ideal methodologies for exploring the direct effects of metabolites, including OAA and TCA intermediates, on mitochondria and intracellular organelles.

3. The real cool novel finding is the interaction of OAA with CHCH3. For a finding this important, I felt evidence was a bit scant. I feel like the interaction should be confirmed using an orthogonal approach (i.e.: affinity purification or similar). Second, the Y axes in figs 6A and 6C are a bit off. Can the authors explain that? Authors should use WT His6-CHCH3 as internal control in 6C, at the very least. A "positive" control in 6A would also be appreciated.

**Response**: Thank you again for these excellent suggestions! As you suggested, we have re-performed the OAA binding assays using His_6_-tagged full-length CHCH-3 and the N129A and E160A mutants. In MST assays, His_6_-CHCH-3 binds with OAA with *K*_d_=2.34 ± 0.25 μM (**Fig 6. C**), which is similar to the data shown in **Fig 6. A**. In contrast, the CHCH-3(N129A) and (E160A) mutants essentially had no marked binding with OAA (**Fig 6. C**). We have now made the *y*-axis consistent in **Fig 6. A** and **C**. As you pointed out, it will be ideal to use another assay to confirm the interaction of OAA with CHCH-3. Unfortunately, we were not able to achieve the conjugation of OAA to beads for affinity purification, owing to the limited time required for revision. Nevertheless, we hope that you will agree with us that the binding of OAA to CHCH-3 is specific, based on our results that IMMT-1 and CHCH-3(N129A) and CHCH-3(E160A) mutants did not have marked binding with OAA in the same MST assays. We have now included the revised **Fig 6. A** and **C** (please see below) in the revised figures.

As you suggested, we sought to find a “positive” control for the results shown in **Fig 6. A**. Given that OAA is the substrate of PCK-1 and PCK-2, we examined its binding with these two proteins using MST assays. We found that the binding of OAA with PCK-1 (*K*_d_: 331.97 ± 46.11 μM) or PCK2 *K*_d_: 496.37 ± 75.19 μM) was much weaker than the binding with CHCH-3. We have now included these results in **S6 Fig. C** (please see below) and provided description in the revised manuscript.

Minor concerns:

4. - some images are puzzling and do not really align with what authors say in the text. Ex: Fig 1I: the deltaMTS mutant (GFP) is not visible. Fig 5C: the chch-3:GFP mutant is also not visible.

**Response**: Thank you very much for pointing out these flaws. We have now provided clearer image for hPCK2(ΔMTS)::GFP in **Fig 1. I** (please see below) and in imunostained CHCH-3 expression (which was suggested by the other reviewer) in **Fig 5. C** (please see below). We also examined other images in all figures to make sure that they are of high resolution.

5. - in general, the "human" data in Fig 7 are cool but a bit poor. As I said, I don't understand the rescue with extracellular OAA. In addition, the specificity of the drug (MPA) for the mitochondrial isoform is in question.

**Response**: Thank you for appreciating the results in Fig. 7, which show that OAA accumulation, either by chemical inhibition of human PCK1 and PCK2 or by directly supplementation of OAA to cells, caused mitochondrial damage as in *C. elegans*. Like in *C. elegans*, reinforced expression of MIC60 or MIC19 provided cells with resistance to OAA-induced mitochondrial damage. These findings, together with other data in our study, suggest that OAA damage mitochondria through a conserved mechanism.

Regarding the specificity of 3-MPA (3-Mercaptopicolinic acid), previous crystal structural analysis revealed that 3-MPA has two binding pockets in PCK1 (Balan MD. et al., **Biochemistry**. 2015; 54(38):5878-5887. DOI: **[10.1021/acs.biochem.5b00822](https://doi.org/10.1021/acs.biochem.5b00822)**). In several other studies, 3-MPA was used as the inhibitor of both the cytoplasmic PCK1 and mitochondrial PCK2 in mammalian cells (e.g. **PMID: 26322521**, PMID: 4**0235441**, PMID: **33335245)**. Thus, we used 3-MPA to inhibit human PCKs in this study and found it indeed caused the elevation of OAA levels in cells and mitochondria.

**Reviewer #2:**

Summary:

In this study Zhang and colleagues provide a mechanistic understanding of mitochondrial defects observed in phosphoenolpyruvate carboxykinase (PCK) mutants of C. elegans, which were first identified through a genetic screen. The authors show that animals with PCK-1 and PCK-2 mutations display a very severe mitochondrial phenotype characterised by a fragmented, enlarged and spherical mitochondrial shape. Through mutations of other genes acting in oxaloacetate metabolism and direct feeding of C. elegans animals with the metabolite, they prove that this is very certainly a direct effect of oxaloacetate accumulation. By overexpressing genes of various known regulators of mitochondrial morphology, the authors show that the MICOS components MIC60 and MIC19 (IMMT-1 and CHCH-3 in C. elegans) can rescue the defects observed in oxaloacetate-accumulating mutants. They further prove that oxaloacetate has a similarly adverse effect on mitochondrial morphology in human cells with a comparable set of experiments, showing that this must be a general effect of oxaloacetate on metazoan mitochondria. In in vitro assays, the authors try to gain a molecular understanding of their cellular observations. They find that oxaloacetate directly binds to the CHCH domain of CHCH-3 and may reduce the protein's ability to promote membrane deformations induced by IMMT-1.

While the link between elevated levels of oxaloacetate and disturbed mitochondria is compelling, we believe that more evidence is required to clearly state that oxaloacetate damages mitochondria by perturbing Mic60-dependent membrane remodeling. We also make suggestions on which controls could be employed to make a stronger case for such an interpretation.

**Response:** We are glad that you appreciated our findings, and we thank you very much for making constructive suggestions! Following these suggestions we have performed additional experiments and supplied the new data in our revision, which greatly improved the quality of our paper. Listed below are our point-by-point responses to your comments and suggestions.

**Major points**:

1. From the evidence provided, it is clear that the MIC60 subcomplex plays a role in rescuing mitochondrial morphology during oxaloacetate accumulation. However, to provide evidence that oxaloacetate exerts its effect directly through binding of MIC19, as suggested by the authors, the CHCH-3(N129A) and CHCH-3(E160A) mutations inhibiting oxaloacetate binding should be introduced in vivo. This would show whether oxaloacetate acts primarily through MIC19 binding.

**Response:** Thank you very much for these excellent suggestions! As you suggested, we overexpressed CHCH-3(N129A) and CHCH-3(E160A), with no tag, in *pck-2;pck-1* double mutants. Unlike wild-type CHCH-3, these mutant proteins failed to restore the spherical and fragmented mitochondria in *pck-2;pck-1* double mutants to wild-type tubular morphology. The untagged CHCH-3 and mutant proteins were visualized by immunostaining. These new data are now presented in the new **Fig 6. D** (please see below).

We also performed in vitro liposome assays, and found that incubation of CHCH-3(N129A) and CHCH-3(E160A) with IMMT-1 failed to promote IMMT-1-dependent branching of membrane tubules. These new data are now presented in the new **Fig 6. E** (please see below). Together these new in vivo and in vitro results suggest that oxaloacetate (OAA) binding inhibits the function of CHCH-3 to promote IMMT-1-dependent membrane shaping.

2. An alternative explanation for the ameliorated mitochondrial morphology upon upregulation of CHCH-3/MIC19 during oxaloacetate accumulation (Figure 5A and B, Figure 7G) would be the sequestration of oxaloacetate, rather than the direct action of CHCH-3/MIC19. Therefore, we suggest that the authors check the protein levels of CHCH-3 or MIC19 in all experiments where IMMT-1 or MIC60 is overexpressed in C. elegans, or human cell lines.

**Response**: Thank you again for these excellent suggestions! As you suggested, we examined the expression of CHCH-3 in *C. elegans* and MIC19 in mammalian cells when overexpressing IMMT-1 or MIC60. Our data indicated that overexpressing IMMT-1::GFP in worms did not markedly affect CHCH-3 levels (please see below in **Fig. A**), and overexpression of MIC60-EGFP or MIC19-Flag in mammalian cells did not markedly affect endogenous MiC19 or MIC60 (Please see below in **Fig 7. G**). To make the manuscript succinate, we have only presented the new **Fig 7. G** (please see below) in the revised manuscript.

3. Connected to the above point: when MIC19-EGFP is overexpressed in U2OS cells, it does not co-localise with mitochondria (Figure 7G). This, at least, needs to be discussed by the authors in the manuscript, as the rescue cannot be due to the canonical function of the protein at cristae membranes in this case. Since this is an important issue for the main claim of the manuscript the authors should try to address this issue experimentally. The miss-localisation might be due to the fluorescent tag at the c-terminus. Tagging Mic19 at the n-terminus might not solve the problem. N-terminal tagging prevents myristoylation and this might lead to import defects. The authors could instead overexpress an untagged version of the protein. Overexpression should be confirmed via western blot and localisation can be followed via IF on fixed cells.

**Response**: These are excellent suggestions regarding the localization of MIC19. As you suggested, we overexpressed Flag-tagged MIC19 and examined its localization by immunostaining using Flag antibody. The expression levels were examined by western blotting with Flag antibody. Our new data showed that overexpressed MIC19-Flag indeed localized to mitochondria, and its overexpression conferred resistance to mitochondrial damage by 3-MPA or OAA. In addition, overexpression of MIC60-EGFP did not markedly affects the protein level of endogenous MIC19. Likewise, overexpression of MIC19-Flag did not affect the expression level of endogenous MIC60. We have now presented these new data in the new **Fig 7. F** and **Fig 7. G** (please see below).

4. The method section describing protein purification of CHCH-3 and IMMT-1 should be more detailed in order for the readers to be able to replicate the experiment, but also to be able to understand the experimental set-up of the in-vitro assays used. Specifically, information about whether full-length proteins, or only certain protein domains were expressed should be provided. Moreover, information on the buffers used during purification is missing. As IMMT-1 is a transmembrane protein the authors should also comment on the solubility of the protein during purification and whether any detergents were used to keep the protein in solution.

In previous liposome tubulation assays with fungal homologues of IMMT-1, a transmembrane domain-lacking version of the protein incubated with liposomes (Ref. 39), or the full-length protein was incorporated into the liposome membrane (Ref. 37) prior to electron microscopy imaging. It should be clarified, whether IMMT-1 was incorporated into the liposome membrane, whether the purified IMMT-1 was soluble, and whether detergents have been employed.

**Response**: These are indeed very important points! To clarify these points, we optimized our protein purification assays, and re-performed the liposome assays. The results, which are consistent with our previous data in the original manuscript, are now presented in our revised manuscript. With these new experimental data, we have made clear the following points: 1) We expressed and purified the full-length IMMT-1. 2) In our new experiments, we used a typical lysis buffer that is used for membrane purification. This new lysis buffer contains 50 mM HEPES, pH 7.4, 150 mM NaCl, 10% glycerol, and 1% n-dodecyl-β-D-maltoside (DDM), 1 mM PMSF. DDM is the primary detergent for solubilizing membrane proteins, which we used all although the purification process. DDM was finally removed from the purified protein solution (50 mM HEPES, pH 7.4, 150 mM NaCl) by using a Millipore Amicon Ultra ultrafiltration tube. We have now provided the details of protein purification in the revised **Methods** section. 3) With this protein purification methods, we obtained soluble and stable IMMT-1. The liposomes were prepared in the same buffer (50 mM HEPES, pH 7.4, 150 mM NaCl) of proteins. Because the buffer did not contain any detergent, the liposomes we prepared were intact, and they were tubulated by IMMT-1. These details are also outline in the revise **Methods**.

5. The interpretation of the membrane moieties accumulating along liposome tubules as branching of the tubule (Figure 6E) needs to be better evidenced. From the micrographs it is unclear if the lipid tubule has a continuous lumen with these moieties, which could indicate branching, or whether these structures are fully separated by membranes. The authors could choose a dye (eg. a DAB-based reaction) visible in EM to fill the lumen of the liposomes and show that the lumen between the tubules and "branches" is continuous. Alternatively, they could provide volume-EM data on these structures to prove that CHCH-3 has an effect on membrane shaping, including branching of tubules.

**Response**: Thank you for these constructive suggestions! As you suggested, we re-performed the liposome assays using the better purified IMMT-1 and CHCH-3 proteins, and used improved EM assays to visualize the membrane shaping by IMMT-1 in the absence or presence of wild-type or mutant CHCH-3. Our new experimental results clearly showed that IMMT-1, but not CHCH-3, tubulated liposomes. When CHCH-3 was incubated with IMMT-1, they generated membrane tubules with branches and button-like structures. These data are consistent with the previous data in our original manuscript. Importantly, with the improved EM-liposome assays, we can see that these membrane tubules and branches are continuous (please see below in **Fig 6. E**). Importantly, CHCH-3(N129A) and CHCH-3(E160A) failed to promote IMMT-1-dependent formation of membrane branches and button-like structures (please see below in **Fig 6. E**). Furthermore, adding OAA, but not Asp or Mal, inhibited the function of CHCH-3 to promote IMMT-1-dependent membrane branches and buttons (please see below in **Fig 6. F**). Altogether, these results support the conclusion that OAA acts through CHCH-3 to inhibit IMMT-1/Mic60-dependent membrane shaping. We have now presented these new results with quantifications in the new **Fig 6. E** and **Fig 6. F** in our revised manuscript. We hope you will agree with us on these revisions.

Minor points:

6. Throughout the manuscript, the authors describe the organisation of mitochondrial cristae as disrupted in certain C. elegans mutants or in OAA-fed animals (lines 179, 278, 293, 352). While we can appreciate that there is a very severe phenotype in mitochondrial morphology related to the fragmentation, size and shape of mitochondria, we feel that the analysis of the phenotype with regards to cristae morphology could be more specific and quantitative. From the TEM images provided (Figures 2B, 4C, 4K), the crista membranes of mutants look similar to those of WT animals and the difference lies rather in the matrix volume than in cristae morphology. If the authors want to make the point of changed cristae organisation, it would be helpful to provide a quantification of specific parameters such as cristae (-junction) number per length of inner mitochondrial membrane, cristae length, or cristae spacing.

**Response**: Thank you very much again for these excellent suggestions! Following your suggestions, we have quantified the number of cristae in mitochondria observed by TEM. These new data are now presented in the new **Fig 2. B, Fig 3. F, Fig 4. C, Fig 4. K, Fig 5.B, and S5 Fig. B** (please see below and the revised figures). We also provided description of these features in the revised manuscript.

7. In the analysis of the TEM images of human mitochondria (Figure 7E), a quantification of aberrant mitochondrial morphology is provided. However, also here the description of the disruption of cristae structure and its quantification could be more specific.

**Response**: This is another excellent point! As you suggested, we have now quantified the numbers of mitochondrial cristae and crista junctions (CJs) in the TEM images in **Fig 7. E** (please see below).

8. Typo: Lactic Academia (line 81)

**Response**: Many thanks for pointing out this typo. This has been corrected now.
