# Supplementary material for: Oxaloacetate damages mitochondria by perturbing MIC60-dependent membrane remodeling
Source: PLoS Biol. 2026 Aug 28;24(8):e3003649. doi: 10.1371/journal.pbio.3003649 (PMC13537684; doi:10.1371/journal.pbio.3003649)
Supplement: Peer review history

# Editor report: Decision Letter 0

**DOI:** 10.1371/journal.pbio.3003649.r001

**Authors:** Ines Alvarez-Garcia

26 Jan 2026

Dear Dr Yang,

Thank you for submitting your manuscript entitled "Oxaloacetate damages mitochondria by perturbing MIC60-dependent membrane remodeling" for consideration as a Research Article by PLOS Biology.

Your manuscript has now been evaluated by the PLOS Biology editorial staff as well as by an academic editor with relevant expertise and I am writing to let you know that we would like to send your submission out for external peer review.

However, before we can send your manuscript to reviewers, we need you to complete your submission by providing the metadata that is required for full assessment. To this end, please login to Editorial Manager where you will find the paper in the 'Submissions Needing Revisions' folder on your homepage. Please click 'Revise Submission' from the Action Links and complete all additional questions in the submission questionnaire.

Once your full submission is complete, your paper will undergo a series of checks in preparation for peer review. After your manuscript has passed the checks it will be sent out for review. To provide the metadata for your submission, please Login to Editorial Manager (https://www.editorialmanager.com/pbiology) within two working days, i.e. by Jan 28 2026 11:59PM.

If your manuscript has been previously peer-reviewed at another journal, PLOS Biology is willing to work with those reviews in order to avoid re-starting the process. Submission of the previous reviews is entirely optional and our ability to use them effectively will depend on the willingness of the previous journal to confirm the content of the reports and share the reviewer identities. Please note that we reserve the right to invite additional reviewers if we consider that additional/independent reviewers are needed, although we aim to avoid this as far as possible. In our experience, working with previous reviews does save time.

If you would like us to consider previous reviewer reports, please edit your cover letter to let us know and include the name of the journal where the work was previously considered and the manuscript ID it was given. In addition, please upload a response to the reviews as a 'Prior Peer Review' file type, which should include the reports in full and a point-by-point reply detailing how you have or plan to address the reviewers' concerns.

During the process of completing your manuscript submission, you will be invited to opt-in to posting your pre-review manuscript as a bioRxiv preprint. Visit http://journals.plos.org/plosbiology/s/preprints for full details. If you consent to posting your current manuscript as a preprint, please upload a single Preprint PDF.

Feel free to email us at plosbiology@plos.org if you have any queries relating to your submission.

Kind regards,

Ines

--

Ines Alvarez-Garcia, PhD

Senior Editor

PLOS Biology

ialvarez-garcia@plos.org

# Peer review: Decision Letter 1

**DOI:** 10.1371/journal.pbio.3003649.r002

**Authors:** Ines Alvarez-Garcia

2 Apr 2026

Dear Dr Yang,

Thank you for your patience while your manuscript entitled "Oxaloacetate damages mitochondria by perturbing MIC60-dependent membrane remodeling" was peer-reviewed at PLOS Biology. Please also accept my apologies for the delay in sending you our decision. The manuscript has now been evaluated by the PLOS Biology editors, an Academic Editor with relevant expertise, and by two independent reviewers.

The reviews are attached below. As you will see, the reviewers find the conclusions interesting, but they also raise several concerns that will need to be addressed before we can consider the manuscript for publication. Reviewer 1 thinks that more controls should be added, including the quantification of specific metabolites to show whether OAA accumulation takes place in the cytoplasm or in the mitochondria, or alternative approaches to address this question. In addition, the reviewer asks for confirmation of the direct interaction of OAA with CHCH3 and adding missing controls. Reviewer 2 thinks that the direct effect of OAA by binding MIC19 should be confirmed by testing the CHCH-3(N129A) and CHCH-3(E160A) mutations in vivo, and that the protein levels of CHCH-3 or MIC19 should be quantified in all the experiments overexpressing IMMT-1 or MIC60 in C. elegans or human cells. The reviewer also requests MIC19 overexpression to see if its mis-localisation is due to the fluorescent tag used in the current experiments, and asks for experiments to confirm that the membrane moieties accumulate along liposome tubules as branching of the tubule, along with more details in the methods.

In light of the reviews, we would like to invite you to revise the work to thoroughly address the reviewers' reports. Given the extent of revision needed, we cannot make a decision about publication until we have seen the revised manuscript and your response to the reviewers' comments. Your revised manuscript is likely to be sent for further evaluation by all or a subset of the reviewers.

In addition to these revisions, you will need to complete some formatting changes, which you will receive in a follow up email. A member of our team will be in touch with a set of requests shortly.

We expect to receive your revised manuscript within 3 months. Please email us (plosbiology@plos.org) if you have any questions or concerns, or would like to request an extension.

At this stage, your manuscript remains formally under active consideration at our journal; please notify us by email if you do not intend to submit a revision so that we may withdraw it.

**IMPORTANT - SUBMITTING YOUR REVISION**

Your revisions should address the specific points made by each reviewer. Please submit the following files along with your revised manuscript:

1. A 'Response to Reviewers' file - this should detail your responses to the editorial requests, present a point-by-point response to all of the reviewers' comments, and indicate the changes made to the manuscript.

*NOTE: In your point-by-point response to the reviewers, please provide the full context of each review. Do not selectively quote paragraphs or sentences to reply to. The entire set of reviewer comments should be present in full and each specific point should be responded to individually, point by point.

You should also cite any additional relevant literature that has been published since the original submission and mention any additional citations in your response.

2. In addition to a clean copy of the manuscript, please also upload a 'track-changes' version of your manuscript that specifies the edits made. This should be uploaded as a "Revised Article with Changes Highlighted" file type.

3. Resubmission Checklist

When you are ready to resubmit your revised manuscript, please refer to this resubmission checklist: https://plos.io/Biology_Checklist

To submit a revised version of your manuscript, please go to https://www.editorialmanager.com/pbiology/ and log in as an Author. Click the link labelled 'Submissions Needing Revision' where you will find your submission record.

Please make sure to read the following important policies and guidelines while preparing your revision and fulfil the editorial requests:

a) *PLOS Data Policy*

Please note that as a condition of publication PLOS' data policy (http://journals.plos.org/plosbiology/s/data-availability) requires that you make available all data used to draw the conclusions arrived at in your manuscript. If you have not already done so, you must include any data used in your manuscript either in appropriate repositories, within the body of the manuscript, or as supporting information (N.B. this includes any numerical values that were used to generate graphs, histograms etc.). Please also indicate in each figure legend where the data can be found. For an example see here: http://www.plosbiology.org/article/info%3Adoi%2F10.1371%2Fjournal.pbio.1001908#s5

b) *Published Peer Review*

Please note while forming your response, if your article is accepted, you may have the opportunity to make the peer review history publicly available. The record will include editor decision letters (with reviews) and your responses to reviewer comments. If eligible, we will contact you to opt in or out. Please see here for more details:

https://blogs.plos.org/plos/2019/05/plos-journals-now-open-for-published-peer-review/

c) *Blot and Gel Data Policy*

Please provide the original, uncropped and minimally adjusted images supporting all blot and gel results reported in an article's figures or Supporting Information files. We will require these files before a manuscript can be accepted so please prepare them now, if you have not already uploaded them. Please carefully read our guidelines for how to prepare and upload this data: https://journals.plos.org/plosbiology/s/figures#loc-blot-and-gel-reporting-requirements

d) *Protocols deposition*

To enhance the reproducibility of your results, we recommend that if applicable you deposit your laboratory protocols in protocols.io, where a protocol can be assigned its own identifier (DOI) such that it can be cited independently in the future. For instructions see: https://journals.plos.org/plosbiology/s/submission-guidelines#loc-materials-and-methods

Thank you again for your submission to our journal. We hope that our editorial process has been constructive thus far, and we welcome your feedback at any time. Please don't hesitate to contact us if you have any questions or comments.

Sincerely,

Ines

--

Ines Alvarez-Garcia, PhD

Senior Editor

PLOS Biology

ialvarez-garcia@plos.org

------------------------------------

Reviewers' comments

Rev. 1:

In this manuscript, the authors investigate the impact of oxaloacetate (OAA) accumulation on mitochondrial disfunction in C. elegans, unveiling a physical interaction between OAA and IMMT-1/MIC60 proteins. Using an unbiased genetic screening in C. elegans, they identified two mutants in the pck-2 gene with abnormally spherical and enlarged mitochondria compared to N2 wild-type animals. pck-2 encodes for an enzyme that, together with PCK-1, converts cytosolic OAA into PEP and both pck-1, pck-2 and pck1;pck2 double mutants display disrupted mitochondria, reduced ATP levels, reduced OCR and reduced body length, body bends and lifespan compared to wild-type animals. This phenotype was associated with increased cytosolic and mitochondrial OAA levels and mutation of got-1.2 or silencing of mdh-2 both rescued mitochondrial morphology and normal OAA levels, as these two enzymes respectively produce OAA in the cytosol and in the mitochondria. On the other hand, got-2.1 and got-2.2 mutants are no longer able to convert mitochondrial OAA into Aspartate, leading to augmented mitochondrial OAA levels and to mitochondrial disfunction. Authors then tested the overexpression of different proteins involved in membrane remodeling in pck-1 and pck-2 mutants to find possible players involved in the observed mitochondrial abnormalities. They found out that overexpression of both IMMT-1/MIC60 and CHCH-3/MIC19 restored normal mitochondrial morphology and counteracted the effects of increased OAA levels, with IMMT-1 acting downstream of CHCH-3. Mechanistically, they demonstrated that only CHCH-3 is able to bind OAA and that this binding CHCH-3 function of promoting IMMT-1- dependent membrane shaping and mitochondrial tubulation. Finally, authors showed that these mechanisms are conserved also in human cells, as the pharmacological inhibition of hPCK1 and hPCK2 leads to OAA increase and so to abnormal mitochondrial morphology and this phenotype is rescued by overexpression of both MIC60 and MIC19.

The work presented in this paper is linear and well organized. The rationale behind each experiment is well explained and conclusions drawn generally make sense. In all, this manuscript advances the knowledge of how the elevation of a single metabolite can impact mitochondrial morphology (and so cell functions) acting through a newly discovered MIC19/MIC60-dependend axis. The experiments are presented in a logical flow, however some concerns stand.

My main, fundamental, concern is that authors do not robustly demonstrate whether OAA accumulation occurs in the cytoplasm or in the mitochondria. They quantify both cytosolic and mitochondrial OAA post fractionation, but metabolites diffuse rapidly and their compartmentalization cannot be assessed by standard organelles partitioning. This is very well known. Authors should add more controls to their experiments (e.g.: quantify metabolites that are exclusively mito or cyto, in parallel; or introduce some type of internal standard for organelle separation e.g.:PMID: 34856123). Or use orthogonal approaches that enable spatial resolution.

Authors tend to be very loose in the interpretation of data the point to a specific elevation of the mito OAA pool. First, the original hit (mutant pgk-2) increases cytoplasmic OAA. Second, TCA and GOT enzymes are bi-directional. Third, in the final experimentation (on human cells) rescue is performed through extracellular OAA supplementation, that would feed the cytoplasmic pool and only very indirectly the mito pool. Only to mention a few. I think authors should be more direct in their analysis. At the end of the day, I understand the challenge posed by the matter. But lacking a very robust quantification of metabolite compartmentalization, conclusions must be very judicious.

The real cool novel finding is the interaction of OAA with CHCH3. For a finding this important, I felt evidence was a bit scant. I feel like the interaction should be confirmed using an orthogonal approach (i.e.: affinity purification or similar). Second, the Y axes in figs 6A and 6C are a bit off. Can the authors explain that? Authors should use WT His6-CHCH3 as internal control in 6C, at the very least. A "positive" control in 6A would also be appreciated.

Minor concerns:

- some images are puzzling and do not really align with what authors say in the text. Ex: Fig 1I: the deltaMTS mutant (GFP) is not visible. Fig 5C: the chch-3:GFP mutant is also not visible.

- in general, the "human" data in Fig 7 are cool but a bit poor. As I said, I don't understand the rescue with extracellular OAA. In addition, the specificity of the drug (MPA) for the mitochondrial isoform is in question.

Rev. 2:

Summary:

In this study Zhang and colleagues provide a mechanistic understanding of mitochondrial defects observed in phosphoenolpyruvate carboxykinase (PCK) mutants of C. elegans, which were first identified through a genetic screen. The authors show that animals with PCK-1 and PCK-2 mutations display a very severe mitochondrial phenotype characterised by a fragmented, enlarged and spherical mitochondrial shape. Through mutations of other genes acting in oxaloacetate metabolism and direct feeding of C. elegans animals with the metabolite, they prove that this is very certainly a direct effect of oxaloacetate accumulation. By overexpressing genes of various known regulators of mitochondrial morphology, the authors show that the MICOS components MIC60 and MIC19 (IMMT-1 and CHCH-3 in C. elegans) can rescue the defects observed in oxaloacetate-accumulating mutants. They further prove that oxaloacetate has a similarly adverse effect on mitochondrial morphology in human cells with a comparable set of experiments, showing that this must be a general effect of oxaloacetate on metazoan mitochondria. In in vitro assays, the authors try to gain a molecular understanding of their cellular observations. They find that oxaloacetate directly binds to the CHCH domain of CHCH-3 and may reduce the protein's ability to promote membrane deformations induced by IMMT-1.

While the link between elevated levels of oxaloacetate and disturbed mitochondria is compelling, we believe that more evidence is required to clearly state that oxaloacetate damages mitochondria by perturbing Mic60-dependent membrane remodeling. We also make suggestions on which controls could be employed to make a stronger case for such an interpretation.

Major points:

1.

From the evidence provided, it is clear that the MIC60 subcomplex plays a role in rescuing mitochondrial morphology during oxaloacetate accumulation. However, to provide evidence that oxaloacetate exerts its effect directly through binding of MIC19, as suggested by the authors, the CHCH-3(N129A) and CHCH-3(E160A) mutations inhibiting oxaloacetate binding should be introduced in vivo. This would show whether oxaloacetate acts primarily through MIC19 binding.

An alternative explanation for the ameliorated mitochondrial morphology upon upregulation of CHCH-3/MIC19 during oxaloacetate accumulation (Figure 5A and B, Figure 7G) would be the sequestration of oxaloacetate, rather than the direct action of CHCH-3/MIC19. Therefore, we suggest that the authors check the protein levels of CHCH-3 or MIC19 in all experiments where IMMT-1 or MIC60 is overexpressed in C. elegans, or human cell lines.

2.

Connected to the above point: when MIC19-EGFP is overexpressed in U2OS cells, it does not co-localise with mitochondria (Figure 7G). This, at least, needs to be discussed by the authors in the manuscript, as the rescue cannot be due to the canonical function of the protein at cristae membranes in this case. Since this is an important issue for the main claim of the manuscript the authors should try to address this issue experimentally. The miss-localisation might be due to the fluorescent tag at the c-terminus. Tagging Mic19 at the n-terminus might not solve the problem. N-terminal tagging prevents myristoylation and this might lead to import defects. The authors could instead overexpress an untagged version of the protein. Overexpression should be confirmed via western blot and localisation can be followed via IF on fixed cells.

3.

The method section describing protein purification of CHCH-3 and IMMT-1 should be more detailed in order for the readers to be able to replicate the experiment, but also to be able to understand the experimental set-up of the in-vitro assays used. Specifically, information about whether full-length proteins, or only certain protein domains were expressed should be provided. Moreover, information on the buffers used during purification is missing. As IMMT-1 is a transmembrane protein the authors should also comment on the solubility of the protein during purification and whether any detergents were used to keep the protein in solution.

In previous liposome tubulation assays with fungal homologues of IMMT-1, a transmembrane domain-lacking version of the protein incubated with liposomes (Ref. 39), or the full-length protein was incorporated into the liposome membrane (Ref. 37) prior to electron microscopy imaging. It should be clarified, whether IMMT-1 was incorporated into the liposome membrane, whether the purified IMMT-1 was soluble, and whether detergents have been employed.

4.

The interpretation of the membrane moieties accumulating along liposome tubules as branching of the tubule (Figure 6E) needs to be better evidenced. From the micrographs it is unclear if the lipid tubule has a continuous lumen with these moieties, which could indicate branching, or whether these structures are fully separated by membranes. The authors could choose a dye (eg. a DAB-based reaction) visible in EM to fill the lumen of the liposomes and show that the lumen between the tubules and "branches" is continuous. Alternatively, they could provide volume-EM data on these structures to prove that CHCH-3 has an effect on membrane shaping, including branching of tubules.

Minor points:

Throughout the manuscript, the authors describe the organisation of mitochondrial cristae as disrupted in certain C. elegans mutants or in OAA-fed animals (lines 179, 278, 293, 352). While we can appreciate that there is a very severe phenotype in mitochondrial morphology related to the fragmentation, size and shape of mitochondria, we feel that the analysis of the phenotype with regards to cristae morphology could be more specific and quantitative. From the TEM images provided (Figures 2B, 4C, 4K), the crista membranes of mutants look similar to those of WT animals and the difference lies rather in the matrix volume than in cristae morphology. If the authors want to make the point of changed cristae organisation, it would be helpful to provide a quantification of specific parameters such as cristae (-junction) number per length of inner mitochondrial membrane, cristae length, or cristae spacing.

In the analysis of the TEM images of human mitochondria (Figure 7E), a quantification of aberrant mitochondrial morphology is provided. However, also here the description of the disruption of cristae structure and its quantification could be more specific.

Typo: Lactic Academia (line 81)

# Author comment: Author response to Decision Letter 2

**DOI:** 10.1371/journal.pbio.3003649.r003

15 Jul 2026

Attachment Submitted filename: Response to Reviewers_OAA.docx

# Peer review: Decision Letter 2

**DOI:** 10.1371/journal.pbio.3003649.r004

**Authors:** Ines Alvarez-Garcia

6 Aug 2026

Dear Dr Yang,

Thank you for your patience while we considered your revised manuscript "Oxaloacetate damages mitochondria by perturbing MIC60-dependent membrane remodeling" for publication as a Research Article at PLOS Biology. Please note that I am currently handling your manuscript since my colleague Ines Alvarez-Garcia is out of the office this week. This revised version of your manuscript has been evaluated by the PLOS Biology editors, the Academic Editor and one of the original reviewers.

Based on the review and our Academic Editor's assessment of your revision, I am pleased to say that we are likely to accept this manuscript for publication, provided you satisfactorily address the following data and other policy-related requests that I have provided below (A-D):

(A) Thank you for already providing the original and uncropped gel images in the S1 and S2 Raw Images files. I would be grateful if these files could be combined into a single S1 Raw Images file to streamline the Supplementary Information.

(B) Please ensure that each of the relevant figure legends in your manuscript include information on *WHERE THE UNDERLYING DATA CAN BE FOUND*, and ensure your supplemental data file/s has a legend.

(C) Per journal policy, if you have generated any custom code during the course of this investigation, please make it available without restrictions. Please ensure that the code is sufficiently well documented and reusable, and that your Data Statement in the Editorial Manager submission system accurately describes where your code can be found. More information on our Code Policy, what and how to share can be found here: https://journals.plos.org/plosbiology/s/code-availability

Please note that we cannot accept sole deposition of code in GitHub, as this could be changed after publication. However, you can archive this version of your publicly available GitHub code to Zenodo. Once you do this, it will generate a DOI number, which you will need to provide in the Data Accessibility Statement (you are welcome to also provide the GitHub access information). See the process for doing this here: https://docs.github.com/en/repositories/archiving-a-github-repository/referencing-and-citing-content

(D) Please ensure that your Data Statement in the submission system accurately describes where your data can be found and is in final format, as it will be published as written there.

------------------------------------------------------------------------

As you address these items, please take this last chance to review your reference list to ensure that it is complete and correct. If you have cited papers that have been retracted, please include the rationale for doing so in the manuscript text, or remove these references and replace them with relevant current references. Any changes to the reference list should be mentioned in the cover letter that accompanies your revised manuscript.

In addition to these revisions, you may need to complete some formatting changes, which you will receive in a follow up email. A member of our team will be in touch with a set of requests shortly. If you do not receive a separate email within a few days, please assume that checks have been completed, and no additional changes are required.

We expect to receive your revised manuscript within two weeks.

To submit your revision, please go to https://www.editorialmanager.com/pbiology/ and log in as an Author. Click the link labelled 'Submissions Needing Revision' to find your submission record. Your revised submission must include the following:

- a cover letter that should detail your responses to any editorial requests, if applicable, and whether changes have been made to the reference list

- a Response to Reviewers file that provides a detailed response to the reviewers' comments (if applicable, if not applicable please do not delete your existing 'Response to Reviewers' file.)

- a track-changes file indicating any changes that you have made to the manuscript.

NOTE: If Supporting Information files are included with your article, note that these are not copyedited and will be published as they are submitted. Please ensure that these files are legible and of high quality (at least 300 dpi) in an easily accessible file format. For this reason, please be aware that any references listed in an SI file will not be indexed. For more information, see our Supporting Information guidelines:

https://journals.plos.org/plosbiology/s/supporting-information

*Published Peer Review History*

Please note that you may have the opportunity to make the peer review history publicly available. The record will include editor decision letters (with reviews) and your responses to reviewer comments. If eligible, we will contact you to opt in or out. Please see here for more details:

https://plos.org/published-peer-review-history/

*Press*

Should you, your institution's press office or the journal office choose to press release your paper, please ensure you have opted out of Early Article Posting on the submission form. We ask that you notify us as soon as possible if you or your institution is planning to press release the article.

*Protocols deposition*

To enhance the reproducibility of your results, we recommend that if applicable you deposit your laboratory protocols in protocols.io, where a protocol can be assigned its own identifier (DOI) such that it can be cited independently in the future. Additionally, PLOS ONE offers an option for publishing peer-reviewed Lab Protocol articles, which describe protocols hosted on protocols.io. Read more information on sharing protocols at https://plos.org/protocols?utm_medium=editorial-email&utm_source=authorletters&utm_campaign=protocols

Please do not hesitate to contact me should you have any questions.

Best regards,

Richard

Richard Hodge, PhD

Senior Editor, PLOS Biology

rhodge@plos.org

On behalf of:

Ines Alvarez-Garcia, PhD

Senior Editor, PLOS Biology

ialvarez-garcia@plos.org

------------------------------------------------------------------------

Reviewer remarks:

Reviewer #1: Authors addressed most of my concerns. I do realize some cannot be easily tackled but authors did take my comments seriously

# Editor report: Decision Letter 3

**DOI:** 10.1371/journal.pbio.3003649.r005

**Authors:** Ines Alvarez-Garcia

18 Aug 2026

Dear Dr Yang,

Thank you for the submission of your revised Research Article entitled "Oxaloacetate damages mitochondria by perturbing MIC60-dependent membrane remodeling" for publication in PLOS Biology. On behalf of my colleagues and the Academic Editor, Johannes Herrmann, I am delighted to let you know that we can in principle accept your manuscript for publication, provided you address any remaining formatting and reporting issues. These will be detailed in an email you should receive within 2-3 business days from our colleagues in the journal operations team; no action is required from you until then. Please note that we will not be able to formally accept your manuscript and schedule it for publication until you have completed any requested changes.

Please take a minute to log into Editorial Manager at http://www.editorialmanager.com/pbiology/, click the "Update My Information" link at the top of the page, and update your user information to ensure an efficient production process.

PRESS

We frequently collaborate with press offices. If your institution or institutions have a press office, please notify them about your upcoming paper at this point, to enable them to help maximise its impact. If the press office is planning to promote your findings, we would be grateful if they could coordinate with biologypress@plos.org. If you have previously opted in to the early version process, we ask that you notify us immediately of any press plans so that we may opt out on your behalf.

We also ask that you take this opportunity to read our Embargo Policy regarding the discussion, promotion and media coverage of work that is yet to be published by PLOS. As your manuscript is not yet published, it is bound by the conditions of our Embargo Policy. Please be aware that this policy is in place both to ensure that any press coverage of your article is fully substantiated and to provide a direct link between such coverage and the published work. For full details of our Embargo Policy, please visit http://www.plos.org/about/media-inquiries/embargo-policy/.

Many congratulations and thanks again for choosing PLOS Biology for publication and supporting Open Access publishing. We look forward to publishing your study.

Sincerely,

Ines

--

Ines Alvarez-Garcia, PhD

Senior Editor

PLOS Biology

ialvarez-garcia@plos.org
